# Translational neuronal ensembles: Neuronal microcircuits in psychology, physiology, pharmacology and pathology

**DOI:** 10.3389/fnsys.2022.979680

**Published:** 2022-08-24

**Authors:** Esther Lara-González, Montserrat Padilla-Orozco, Alejandra Fuentes-Serrano, José Bargas, Mariana Duhne

**Affiliations:** ^1^División Neurociencias, Instituto de Fisiología Celular, Universidad Nacional Autónoma de México, Mexico City, Mexico; ^2^Department of Neuroscience, Feinberg School of Medicine, Northwestern University, Chicago, IL, United States; ^3^Department of Neurology, University of California, San Francisco, San Francisco, CA, United States

**Keywords:** neuronal ensembles, neuronal networks, functional connections, synaptic weights, population coding, Parkinson’s disease, L-DOPA induced dyskinesia, epileptiform discharges

## Abstract

Multi-recording techniques show evidence that neurons coordinate their firing forming ensembles and that brain networks are made by connections between ensembles. While “canonical” microcircuits are composed of interconnected principal neurons and interneurons, it is not clear how they participate in recorded neuronal ensembles: “groups of neurons that show spatiotemporal co-activation”. Understanding synapses and their plasticity has become complex, making hard to consider all details to fill the gap between cellular-synaptic and circuit levels. Therefore, two assumptions became necessary: First, whatever the nature of the synapses these may be simplified by “functional connections”. Second, whatever the mechanisms to achieve synaptic potentiation or depression, the resultant synaptic weights are relatively stable. Both assumptions have experimental basis cited in this review, and tools to analyze neuronal populations are being developed based on them. Microcircuitry processing followed with multi-recording techniques show temporal sequences of neuronal ensembles resembling computational routines. These sequences can be aligned with the steps of behavioral tasks and behavior can be modified upon their manipulation, supporting the hypothesis that they are memory traces. In vitro, recordings show that these temporal sequences can be contained in isolated tissue of histological scale. Sequences found in control conditions differ from those recorded in pathological tissue obtained from animal disease models and those recorded after the actions of clinically useful drugs to treat disease states, setting the basis for new bioassays to test drugs with potential clinical use. These findings make the neuronal ensembles theoretical framework a dynamic neuroscience paradigm.

## Introduction

Nephrons, lobules, alveoli, acini and so forth work as functional units in body organs: multicellular organizations coordinating the actions of different cell classes to receive an input and yield and output, modules commonly ordered in tandem in a given organ and being preserved along the vertebrate phylogeny (Bargas and Pérez-Ortega, 2017). One advantage of these modular architecture is obvious: degradation of an organ due to degenerative chronic disorders or age, is a gradual steady decline before a complete failure is reached. The advent of multicellular recording techniques (Brown et al., 2005; Harris, 2005; Dombeck et al., 2009; Dragoi, 2020; Barack and Krakauer, 2021; Ebitz and Hayden, 2021; Lagache et al., 2021) has shown that many areas of the nervous system have a modular organization of neuronal populations, previously shown indirectly with intra-and extra-cellular unitary recordings associated with local field potentials and other population recordings (Brown, 1914; Hubel and Wiesel, 1962; Steriade and Deschenes, 1984; Arshavsky et al., 1997; Mountcastle, 1997; Timofeev and Steriade, 1997; McCormick, 2002; Grillner, 2006; Kiehn, 2006; Casanova and Casanova, 2018; He et al., 2009): groups of neurons that coordinate themselves to work together in several brain areas to perform their functions have been defined defined as “canonical” microcircuits in Shepherd and Grillner (2018). A new advantage for experimentalists is that territorial coordinates of unitary activity while using multielectrode arrays (MEAs), or various neurons themselves using calcium imaging, can be identified, and followed within and without their groups with single cell resolution (Buzsáki, 2004; Kwan, 2008; Shew et al., 2010; Einevoll et al., 2012; Carrillo-Reid et al., 2015; Stringer et al., 2019) and be compared with population recordings. These findings ended a long debate about the functional unit of the nervous system: either the neuron or neuronal populations (for a historical perspective see: Yuste, 2015). In many brain areas, the functional unit are neuronal ensembles: groups of coactive neurons (see Carrillo-Reid and Yuste, 2020).

## Neuronal ensembles: From synaptic to functional connectivity

There are many classes of neurons with their proper biophysical, biochemical and genetical features (e.g., Llinás, 1988; Bean, 2007; Maudsley et al., 2007; Tse and Wong, 2013; Armand et al., 2021), however, in many areas of the brain, immunostaining, *in situ* hybridization, single cell PCR, transcriptomics and whole cell recordings have shown iterative modules accomplishing coordinated tasks (Markram et al., 2015; Shepherd and Grillner, 2018; Assous and Tepper, 2019; Williams and Riedemann, 2021). In a brief simplification, minimal features of a canonical microcircuit are: a group of GABAergic interneurons innervate principal neurons mainly in the dendrites and spines to control afferent inputs and feed-back entries (e.g., low-threshold spiking – LTS; double bouquet cells; Martinotti cells; with similar markers in several brain places: neuropeptide Y – NPY; nitric oxide synthase – NOS; somatostatin – SOM), while other groups of interneurons mainly innervate the perisomatic area and axon hillock to control principal neurons output (e.g., fast-spiking interneurons – FSIs; basket or chandelier cells; the main marker being parvalbumin – PV). Principal cells are surrounded by these interneurons controlling input and output in a consensual trait, plus interconnections between principal neurons and long-range interneurons form feed-forward networks including disinhibition (Tremblay et al., 2016; Traub et al., 2020). Interneurons are minority and, therefore, are shared by groups of principal cells. How many of these microcircuits and neurons conform an ensemble? The basic connectivity is scalable (e.g., from minicolumns to barrels and regions; Staiger and Petersen, 2021). Perhaps recording of thousands of neurons (Stringer et al., 2021) show only the tip of the iceberg. Alternatively, scalability shows similar sequence patterns from the histological scale (dozens of neurons) to the mesoscale (hundreds of neurons). Regular modular elements with similar electrophysiological profiles and markers expression can be found in all cortical mantles including the hippocampus, the striatal circuit, and with slightly different profiles and names the cerebellum (Douglas and Martin, 2004; D’Angelo et al., 2016; Burke et al., 2017). Importantly, they resonate at different frequencies and are main components of cerebral oscillations and rhythms generation (Hutcheon and Yarom, 2000; Buzsaìki and Draguhn, 2004), which could be a way of additional communication besides axons, terminals and volume transmission (Ito and Schuman, 2008; Jahnke et al., 2014).

An important factor to understand the association of particular neuron types and their cuasi-iterative prevalence is of course programmed embryogenesis (Li et al., 2016). In addition, there is the theoretical framework synthesized by Hebb (1949) and later modified with experiments from numerous groups (e.g., Frégnac, 2003; Malenka and Bear, 2004; Caporale and Dan, 2008; Baltaci et al., 2019; Brown and Donald, 2020; Magee and Grienberger, 2020). Although the generic name for these modules may be: “neuronal ensembles” (other names in: Carrillo-Reid and Yuste, 2020), their composition and complete numbers in different contexts is unknown. For example, by identifying and following samples of motor cortical ensembles *in vitro* at histological level it was found that most sampled ensembles have PV + neurons (Serrano-Reyes et al., 2020). Similar experiments must be done with the different kinds of interneurons and principal cells (Markram et al., 2015) to describe their composition and observe their dynamics, since ensembles are composed with inhibitory and principal neurons playing various roles. Due to these cited experiments, to simplify the cellular-synaptic complexity and make sense at the network level, neuronal ensembles, defined as “groups of neurons that show spatiotemporal co-activation” (Yuste, 2015) are commonly linked with “functional connectivities” between recording sites while using multi-recording techniques (Frőhlich, 2016). Dimensional reduction can be used to position ensembles in a neuronal state space (Ebitz and Hayden, 2021). Neurons with correlated firing are sorted together by functional connections and their activity alternates due to connections between ensembles, which can be illustrated as trajectories in low-dimensional space and connectivity graphs (Pérez-Ortega et al., 2016; Calderón et al., 2022). This simplification allows going from the cellular-synaptic level to the emergent properties of recorded networks (Carrillo-Reid et al., 2015): in a “bottom-up” direction.

## Neuronal ensembles as functional units

The modified Hebbian theoretical framework tries to fill the gap between the cellular-synaptic level and the network level at different scales (Grillner and Graybiel, 2006; Buzsáki, 2010; Tognoli and Kelso, 2014). Beginning with correlated plasticity that can be extended to other plasticity types, it is postulated that correlated or timed activity between pre- and post-synaptic elements reinforce synaptic connections, while decorrelated or inverse timing between these elements weaken synaptic connections. These mechanisms generate long lasting changes in the synaptic weights of the network, so that some synapses are enforced [long-term potentiation (LTP)] and other are debilitated or disconnected long-term depression (LTD), producing preferent paths for the flow of activity, being this mechanism the basis to make new circuits for learning and making memory traces (Kandel et al., 2014; Dringenberg, 2020; Stacho and Manahan-Vaughan, 2022). Besides the unique specialized fast acting synapses that connect neuron groups using postsynaptic ligand-gated ion channels (Cockcroft et al., 1990; Ortells and Lunt, 1995; Auerbach, 2013), alternative communicating pathways - shared with non-excitable cells - are also present: e.g., volume transmission (paracrine communication), extra-synaptic and synaptic receptors coupled with G-proteins (GPCRs) that can or cannot form heteromeric complexes igniting diverse intracellular signaling cascades (e.g., Agnati et al., 1994, 2006; Lefkowitz, 2000; Rasmussen et al., 2011; Latek et al., 2012; Fuxe et al., 2013; Tse and Wong, 2013), plasticity of GABAergic inhibitory neurons (Rueda-Orozco et al., 2009; Castillo et al., 2011; Roth and Draguhn, 2012; Barberis, 2020), anti-Hebbian mechanisms, retrograde signaling, the role of neuromodulators, instructive signals and eligibility traces, different synaptic receptor types and sub-units, etcetera, have increased the complexity of synaptic plasticity (Lamsa et al., 2007; Sjöström et al., 2008; Conde et al., 2013; Piochon et al., 2013; Johansen et al., 2014; Park et al., 2014; Ruan et al., 2014; Gerstner et al., 2018; Langille and Brown, 2018; Cingolani et al., 2019; Bannon et al., 2020; Magee and Grienberger, 2020; Speranza et al., 2021). Countless experiments demonstrate diverse mechanisms for LTP and LTD (e.g., Stanton, 1996; Citri and Malenka, 2007; Kandel et al., 2014; Berry and Nedivi, 2016; Fauth and Tetzlaff, 2016; Abraham et al., 2019; Baltaci et al., 2019; Stampanoni-Bassi et al., 2019; Magee and Grienberger, 2020; Mateos-Aparicio and Rodríguez-Moreno, 2020), modifying the original Hebbian principle (Markram et al., 1997; Martin and Morris, 2002; Nicoll and Roche, 2013; Dringenberg, 2020). Whatever the mechanisms for generating LTP and LTD (Dudek and Bear, 1992; Malenka and Bear, 2004; Caporale and Dan, 2008; Hawes et al., 2013; Kandel et al., 2014; Nicoll, 2017; Diering and Huganir, 2018; Abraham et al., 2019; Brown and Donald, 2020), preferred paths for the flow of activity form stable circuits due to changes in synaptic weights. In turn, stable circuits encode memory traces (Kandel and Schwartz, 1982; Spatz, 1996; Sweatt, 2016; Andersen et al., 2017). It has been demonstrated that connections already set and stable, can be followed along several days (Pérez-Ortega et al., 2021), and that the flow of activity through the network, observed as synchronous or correlated neuronal firing in neuron clusters that alternate their activity in a recurrent or reverberant fashion forming temporal sequences are their manifestation (Beggs and Plenz, 2003; Carrillo-Reid et al., 2008; Rabinovich M. et al., 2008; Rabinovich M. I. et al., 2008; Buonomano and Maass, 2009; Buzsáki, 2010; Plata et al., 2013a,b; Lara-González et al., 2019; Serrano-Reyes et al., 2020). Activity sequenecs, transitions or trajectories between neuronal ensembles, are similar to computational routines that can be recorded at different scales using multi-recording techniques. When recorded *in vivo* in trained animals, ensembles sequences can be aligned with the different steps of a behavioral task (Dombeck et al., 2009; Adler et al., 2012; Bakhurin et al., 2016; Haegens et al., 2017; Jennings et al., 2019; Sheng et al., 2019; Carrillo-Reid and Yuste, 2020; Lagache et al., 2021; Coss et al., 2022; Pimentel-Farfan et al., 2022). Temporal ensembles sequences can be observed during learning and the action of neuromodulators, by following particular identified neurons with calcium imaging, and exciting or inhibiting them with optogenetic techniques (Bliss and Gardner-Medwin, 1973; Carrillo-Reid et al., 2009a,b, 2016, 2019; Grewe et al., 2017; Adler et al., 2019; Josselyn and Tonegawa, 2020). One can hypothesize what are the cellular mechanisms of ensemble alternation, ignition, inhibition, codification of stimulus, actions or decisions, defining possible causal relations with behavior (Takehara-Nishiuchi, 2022). Hypotheses can be tested going back to the cellular-synaptic level and ask for a precise machinery: a guided “top-down” direction. For instance, the original Hebbian proposal forming ensembles (correlative plasticity: “neurons that fire together wire together” – Löwel and Singer, 1992) has received definitive experimental demonstration in the primary visual cortex with optogenetic techniques (Carrillo-Reid et al., 2016). On the other hand, it has been demonstrated that the inhibition of a targeted ensemble with chemogenetic techniques, at the time it enters into the behavioral sequence, does interrupt the learned task (Sheng et al., 2019). Going “blind” at the cellular-synaptic level may discover many interesting phenomena whose importance is only speculative since their actions on the neuronal population are unknown.

## Neuronal ensembles in brain slices: Asking brain tissue what it can do

*In vitro* preparations were originally designed to ask questions at the cellular-synaptic level, however, given the simplifications stated above, one can assume that stable modules can be seen in a piece of tissue isolated from the rest of the system as long as there is a way to turn them on. This was first achieved in the lamprey (Grillner and El Manira, 2020) using targeted and population recordings to observe that the same circuit recorded *in vivo* could be recorded *in vitro*, where drugs concentrations and experimental manipulations are at hand (Grillner and Zangger, 1979; Kiehn, 2006; Carrillo-Reid et al., 2008; Plenz, 2012; Guzulaitis and Hounsgaard, 2018). In many cases, the “switch” to turn on ensemble dynamics was NMDA added to the bath saline, although other chemicals and procedures can be used (Fellous and Sejnowski, 2000; Aparicio-Juárez et al., 2019). In slices, low NMDA concentrations “titrate” the most enforced connections within and between neuronal ensembles and induce their activation (Carrillo-Reid et al., 2008) with the help of the “driving force” given by extra-synaptic receptors (Garcia-Munoz et al., 2015). In this way, recurrent transitions between ensembles that alternate their activity become evident (Carrillo-Reid et al., 2009a). The sole presence of these sequences (once their stochastic appearance has been discarded), that resemble computational routines, suggests that the tissue is storing these trajectories in the absence of long-range connections. In fact, they can be recovered in *ex vivo* slices after learning a task *in vivo* (Yin et al., 2009). They can be identified, quantified and followed through several methods to show the action of neuromodulators (Carrillo-Reid et al., 2009a,^2011^) and pathological states (Jáidar et al., 2010). But many more issues need investigation, for example: what happens with stable sequences and networks after LTP and LTD protocols usually applied to individual synapses.

## Neuronal ensembles in pathological brain tissue

This work emphasizes the histological scale since biopsies have been a main tool in clinical practice to reach diagnosis and prognosis. This makes brain slices a potential translational preparation: bringing the possibility to have living tissue to do functional histology benchside, or even besides the surgery room. Living tissue can be asked whether it can show ensemble sequences or networks that correlate with pathology (Cardin et al., 2010; Rickgauer et al., 2014; Emiliani et al., 2015; Carrillo-Reid et al., 2016; Parker et al., 2018; Peña-Rangel et al., 2021). For instance, pre-clinical investigation shows that while control striatum exhibits a definite sequence of neuronal ensembles (Carrillo-Reid et al., 2008), the parkinsonian striatum (dopamine depleted) shows ensembles stuck in highly recurrent sequences becoming an observational metaphor of patients‘ immobility: akinesia, hypokinesia and rigidity (Jáidar et al., 2010, 2019; Plata et al., 2013a,b; Pérez-Ortega et al., 2016). L-DOPA induced dyskinesia showed more frequent and complex ensemble transitions than in the control, and a persistent recurrence due to the underlying parkinsonism (Pérez-Ortega et al., 2016; Calderón et al., 2022), again, being a tissue “fingerprint” of patients with abnormal involuntary movements. In the motor cortex ensemble sequences accompanied by interneurons in the control, get reduced to two main recurrent ensembles with pyramidal cells and interneurons separated during epileptiform discharges (Serrano-Reyes et al., 2020). After identifying characteristic changes in pathological preparations, it was logical to ask the tissue whether it can show circuit changes induced by drugs of clinical use to treat diseased states.

## Testing drugs with therapeutic potential

*In vitro* preparations can be used as bioassays to test clinical useful drugs using the neuronal ensembles framework. For example, dopamine agonists have been tried to replace or be adjuvants to L-DOPA in the treatment of Parkinson’s disease. Many ligand-binding and biochemical studies have been summarized in an encompassing meta-analysis that classified these drugs in order of efficiency (Millan, 2010). It was found that basically the same order of efficiency is encountered when testing dopamine agonists by using the analyses of ensemble sequences (Lara-González et al., 2019). Vectorizing the properties of ensembles sequences and networks, principal component analysis revealed differences between two widely used anti-dyskinetic drugs (Calderón et al., 2022). Other clinically relevant drugs and disease states need to be tested to standardize this methodology. Standardization may lead to the testing of novel drugs with potential therapeutic use with greater efficacy and in lesser time.

## Concluding remarks

Multi-recording techniques have confirmed that neuronal circuits in many brain areas are composed by neuronal ensembles that alternate their activity forming defined and recurrent temporal sequences. By assuming that the complexity of neuronal connectivity can be represented by functional connections and that the synaptic weights that instantiate these circuits are relatively stable, one can observe these temporal sequences as the manifestations of memory traces following computational routines that can be recorded *in vitro* and *in vivo*. Techniques of neuronal populations analysis are being developed to quantify, follow and decodify these computations. Recordings *in vivo* can be aligned with behavioral tasks and can be manipulated opto- and chemo-genetically to alter behavior showing causality. Recordings *in vitro* can show pathological changes from animal models as well as the actions of clinically or potentially useful drugs and therapeutical procedures. Because this experimental paradigm is at its beginnings, standardization and consensual definitions are still lacking. However, the sole observation of neuronal ensembles trajectories, formation and dissolution, opens many questions for future research: what are the precise mechanisms at the cellular-synaptic level, how these circuits can be manipulated, how can be simulated or modeled by artificial neural networks of diverse complexity, what are the limits of scalability up and down, what is the relation of these phenomena with larger scale observations, e.g., fMRI, what are the brain areas that use this class of population code and what are the areas that do not.

## Author contributions

MD and EL-G wrote the original draft. MP-O and AF-S compilated literature and worked on the first draft. JB revised the drafts and wrote the final version. All authors contributed to the article and approved the submitted version.
